# Supplementary material for: Genome assembly and annotation of the Sharp-nosed Pit Viper Deinagkistrodon acutus based on next-generation sequencing data
Source: GigaByte. 2023 Sep 4;2023:gigabyte88. doi: 10.46471/gigabyte.88 (PMC10498098; doi:10.46471/gigabyte.88)
Supplement: Translation

# Translation: 基于二代测序数据的尖吻蝮基因组的组装与注释

**Authors:** Xinyu Wang, Lirong Liu, Wenbiao Zhu, Shiqing Wang, Minhui Shi, Shuhui Yang, Haorong Lu, Jun Cao

**Affiliations:** ^1^ State Key Laboratory of Agricultural Genomics, Key Laboratory of Genomics, Ministry of Agriculture, BGI Research, Shenzhen 518083, China; ^2^ China National GeneBank, BGI, Shenzhen 518120, China; ^3^ College of Wildlife and Protected Area, Northeast Forestry University, Harbin 150040, China; ^4^ College of Life Sciences, University of Chinese Academy of Sciences, Beijing 100049, China

## Abstract

对目前已知 3,000 余种蛇类的研究可为它们的基因组进化提供有价值的见 解。尖吻蝮，也被称为尖鼻蝮、百步蛇或五步蛇，是一种具有重要经济、医学 和科学价值的毒蛇。其广泛分布于中国东南部和东南亚，主要用于蛇毒研究。 本文采用二代测序技术，组装和注释了一个高度连续的尖吻蝮基因组。基因组 大小为 1.46 Gb; 其 scaffold N50 长度为 6.21 Mb，重复序列含量为 42.81%，共 注释出 24,402 个功能基因。本研究有助于在遗传水平上进一步认识和利用尖吻 蝮及其毒液。

尖吻蝮 (*Deinagkistrodon acutus*) 是属于蛇亚目、蝰科的一种有毒蛇，常被 称为百步蛇、五步蛇、长鼻蝮等（如图1 所示）[1, 2]。其毒液主要具有血液毒 性，可导致凝血功能异常并促进组织损伤、水肿、急性肾衰竭等反应发生，主 要作用于肺部 [3]。尖吻蝮在中国东南部、老挝和越南北部广泛分布，因其较 大的身体以及毒液而具有重要的商用及药用价值 [4, 5]。目前尖吻蝮的研究主 要集中在其毒液的毒性成分、被咬伤患者的症状分析等方面，以及对蛇毒的利 用进行了研究，如体外抑菌、毒液中特定蛋白具有抗血栓、抗凝血活性等 [6–9]。高质量的基因组有助于蛇毒相关基因的发现，进而可以帮助研究人员更好 地了解及利用蛇毒。

**图 1:** 杨典成拍摄的一条尖吻蝮

 本研究基于二代测序数据对尖吻蝮基因组进行组装和注释，这些数据为蛇毒 相关基因的发现及利用、更好地了解蛇的系统发育和进化提供了重要的数据支持。

## 材料与方法

### 样本采集与测序

从安徽省黄山市（中国）获得一条重781 g 的尖吻蝮（NCBI: txid36307）用 于基因组组装及注释。取其肝、胃、肾以及肌肉组织用于 RNA 提取，另取两份 肌肉组织分别用于全基因组测序（Whole Genome Sequencing，WGS）和单管长 片段序列（single-tube long fragment read，stLFR）测序前的 DNA 提取。按照刘 博洋等的方案提取尖吻蝮 DNA、构建文库并进行双端测序（如图 2 所示） [10]。 样本采集及相关实验流程经华大基因机构审查委员会批准（BGI-IRB E22017）。

**图 2:** 收集在 protocols.io 中用于蛇基因组一般测序的说明 [10]. https://www.protocols.io/widgets/doi?uri=dx.doi.org/10.17504/protocols.io.5jyl8j6e9g2w/v2

### 基因组组装 注释及评估

通过 25× WGS 测序数据评估组装的尖吻蝮基因组大小。利用 GCE（v1.0.2, RRID:SCR_017332）的 Kmerfreq 进行 k-mer 频数统计。其输出结果表明共获取 32,372,553,516 个 k-mer 片段（*k* = 19），将上述结果输入 GCE 并使用杂合模式（k-mer 深度峰值为21）评估基因组大小、杂合度等 [11]。我们使用 stLFR 数据、 利用 Supernova（v2.1.1，RRID:SCR_016756）进行基因组组装。为使组装的序列 更完整，利用 GapCloser（v1.12-r6，RRID:SCR_015026）和 WGS 测序数据填补 空白，并利用 redundans（v0.14a）去除基因组的冗余序列 [12]。使用图 2 中描 述的方法获得最终基因组。我们使用从头预测和基于同源性的方法来识别基因组 组装中的重复区域。基于同源性的预测使用 Blastall（v2.2.26）进行 [12]。具体 而言，我们将来自 UniProt 数据库（发布号:2020_05）的东部拟眼镜蛇（Pseudonaja textilis）、虎斑响尾蛇（Crotalus tigris）、束带蛇（Thamnophis elegans）和虎蛇 （Notechis scutatus）的蛋白序列比对到尖吻蝮基因组序列。按照刘博洋等描述的 方案进行基因组注释和评估 [10]。

为重建系统发育树，利用 OrthoFinder（v2.3.7，RRID:SCR_017118）[13] 在 中国林蛙（Rana temporaria，GCA_905171775.1）、古兹沙漠陆龟（Gopherus evgoodei ，GCA_007399415.1）、普通壁蜥（Podarcis muralis ， GCA_004329235.1） 束带蛇（Thamnophis elegans ，GCA_009769535.1）、东部拟眼镜蛇（Pseudonaja textilis ，GCA_900518735.1）蛋白序列中寻找单拷贝同源基因。

## 数据验证和质控

利用 stLFR 测序产生 164.75 Gb 的主要结果文件组装了 1.46 Gb 的尖吻 蝮基因组。基因组的最长和 N50 scaffold 分别为 39.38 Mb 和 6.21 Mb（如表 1 所示），这表明基因组具有高度的连续性。将最终基因组与脊椎动物数据库 （vertebrate_odb10）中 3,354 BUSCOs 进行比较，我们发现在尖吻蝮基因组中， 3,354 个脊椎动物基因有 87.2%，即 2,924 个基因被覆盖；分别仅有 245 个 （7.3%）和 185 个（5.5%）基因部分比对上及未得到比对结果。

**表 1:** 与本研究中组装的尖吻蝮基因组相关的基因组组装数据

Item	Category	Size
	stLFR (Gb) WGS (Gb) RNA-seq (Gb)	164.75 96.76 10.42
Sequencing data	Assembled genome (Gb) Longest Contig (Mb) Contig N50 (Mb) Longest scaffold (Mb) Scaffold N50 (Mb) GC content (%)	1.46 0.52 0.03 39.38 6.21 37.9

尖吻蝮基因组中重复序列总长度为 642 Mb，占整个基因组的 42.81 %（如表 2、图 3 所示）。基于从头预测，统计基因组中各种重复序列含量。最占优势的重 复元件是长散在重复元件（long interspersed nuclear elements ，LINEs）（443 Mb）， 其次是长末端重复（long terminal repeats ，LTRs）（180 Mb）、DNAs（26.43 Mb）和短散在重复元件（short interspersed nuclear elements ，SINEs）（0.94 Mb）。LINEs 和 LTRs 的含量分别为 29.53 % 和 11.99 %（如表 3 所示）。重复序列对于遗传信 息的自我复制具有重要意义，其与物种的遗传和变异息息相关。

**图 3:** 尖吻蝮基因组转座元件（transposable elements ，Tes）的分布。TEs 包括 DNA 和 RNA 转座子（即 DNAs、LINEs、LTRs 和SINEs）。(a) de novo 序列的差异率分布。(b) 已知序列 的差异率分布。

**表 2:** 尖吻蝮基因组重复序列统计

Type	Repeat Size	% of genome
Trf	49,665,678	3.158437
RepeatMasker (RRID:SCR_012954)	254,179,490	16.16428
Proteinmask	190,282,517	12.100819
De novo	636,067,480	40.45005
Total	673,253,494	42.814856

**表 3:** 尖吻蝮基因组重复序列 (*de novo*) 统计

Type	Length (Bp)	% in genome
DNA	27,712,037	1.762318
LINE	464,343,121	29.529418
SINE	984,426	0.062604
LTR	188,498,215	11.987348
Other	0	0
Satellite	1,180,615	0.07508
Simple_repeat	2,250,205	0.143099
Unknown	2,609,514	0.165949
Total	636,067,480	40.45005

共有 24,402 个功能基因被注释 (如表 4 所示)。对功能基因进行基因本体论 (gene ontology, GO) 富集分析结果显示, 基因富集于生物过程 (biological processes, BP)、细胞成分 (cellular components, CC) 和分子功能 (molecular functions, MF) 中。其中细胞过程 (cellular process)、膜 (membrane) 和结合 (binding) 分别在 BP、CC 和 MF 中含量最高。对功能基因进行 KEGG 通路富 集分析结果表明, 信号转导相关基因在尖吻蝮中具有至关重要的作用 (如图 4 所示)。此外, 与代谢相关的富集通路数量最多。

**图 4.** 尖吻蝮基因组的基因注释。(a) GO 富集。(b) KEGG 富集。

构建系统发育树结果 (如图 5 所示) 表明, 我们的数据可以用于物种系统发 育树的构建且该树与其他人的研究结果一致 [14]。通过将本研究中组装的基因 组数据与染色体水平的尖吻蝮基因组数据 [1] 比较可知, 我们成功组装并注释 一个高度连续的尖吻蝮基因组。

**图 5.** 利用核基因组单拷贝基因重建系统发育树。数字表示分支的长度。有色方块表示 bootstraps/metadata, 显示范围为 0.49744 到 1。

**表 4:** 尖吻蝮基因组功能注释结果

	Number	Percentage (%)
Total	24,402	100
Swiss-Prot annotated	19,527	80.02
KEGG annotated	20,869	85.52
TrEMBL annotated	22,927	93.96
InterPro annotated	23,089	94.62
GO annotated	14,512	59.47
Overall	23,844	97.71

## 重用潜力

我们的数据可为其他人研究尖吻蝮提供参考基因组。此外,它也可与其它蛇类基 因组结合使用,用于研究蛇类的系统发育和进化。最后, 我们的基因组可为蛇毒 和相关毒理学研究提供数据支持。

## 发表同意

不适用

## Data Availability

支持本研究结果的数据已存入国家基因库数据库 (CNGBdb) [15] 的 CNGB 序 列档案 (CNSA) [16] 编号为 CNP0004047。原始数据也可通过 PRJNA955401 在 SRA 中获得。其它数据可在 GigaDB 存储库中获得 [17]。

## 缩略词表

BP, biological process; CC, cellular component; GO, gene ontology; LINE, long interspersed nuclear element; LTR, long terminal repeat; MF, molecular function; SINE, short interspersed nuclear elements; stLFR, single-tube long fragment read; TE, transposable elements; WGS, Whole Genome Sequencing.

## 声明

### 伦理批准

样本采集及相关实验流程经华大基因机构审查委员会批准 (BGI-IRB E22017)。

### 作者贡献

曹军、卢浩荣和刘力榕设计并发起了该项目。蛇的样本由安徽师范大学提供。朱 文标和杨淑慧对采集的样本进行处理。王心雨、施敏辉、汪诗晴进行 DNA 提取、 文库构建。王心雨进行数据分析并撰写论文。所有作者都已阅读并同意最终手稿。

### 资助

本项目由广东省高通量基因组测序与合成编辑应用重点实验室资助 (grant no. 2017B030301011), 本研究也得到国家基因库 (CNGB) 的支持。

## References

[1] YinW, WangZ-j, LiQ-y Evolutionary trajectories of snake genes and genomes revealed by comparative analyses of five-pacer viper. Nat. Commun., 2016; 7(1): 13107. doi:10.1038/ncomms13107.27708285PMC5059746

[2] TanKY, ShamsuddinNN, TanCH. Sharp-nosed Pit Viper (*Deinagkistrodon acutus*) from Taiwan and China: A comparative study on venom toxicity and neutralization by two specific antivenoms across the Strait. Acta Trop., 2022; 232: 106495. doi:10.1016/j.actatropica.2022.106495.35504314

[3] HuangJ, ZhaoM, XueC Analysis of the composition of *Deinagkistrodon acutus* snake venom based on proteomics, and its antithrombotic activity and toxicity studies. Molecules, 2022; 27(7): 2229. doi:10.3390/molecules27072229.35408629PMC9000436

[4] HuangF, ZhaoS, TongF Unexpected death in a young man associated with a unilateral swollen leg: Pathological and toxicological findings in a fatal snakebite from *Deinagkistrodon acutus* (Chinese moccasin). J. Forensic Sci., 2021; 66(2): 786–792. doi:10.1111/1556-4029.14622.33227152

[5] WangD-Q, PanL-L, YangD-C Complete mitochondrial genome of the sharp-snouted pitviper *Deinagkistrodon acutus* (Reptilia, Viperidae). Mitochondrial DNA. Part B, Resources, 2019; 4(2): 2900–2901. doi:10.1080/23802359.2019.1660593.33365782PMC7706857

[6] HuX-Q, WuQ-L, LiX-Y Study on venom protein components of *Deinagkistrodon acutus* living in different geographical units. Oxid. Commun., 2016; 39(A2): 1885–1895.

[7] LinfengW, LutaoX, PinL Radial artery aneurysm formation and spontaneous rupture after snake bite to the right forearm. Toxicon, 2020; 181: 79–81. doi:10.1016/j.toxicon.2020.04.098.32360670

[8] HuangJ, SongW, HuaH Antithrombotic and anticoagulant effects of a novel protein isolated from the venom of the *Deinagkistrodon acutus* snake. Biomed. Pharmacother., 2021; 138: 111527. doi:10.1016/J.BIOPHA.2021.111527.33773469

[9] HuangZ, HeD, LiaoM. Antibacterial activity of venoms from Guangxi cobra, *Bungarus multicinctus* and *Deinagkistrodon acutus* in vitro. Chinese J. Microecology, 2019; 31(10): 1135–1139. doi:10.13381/j.cnki.cjm.201910004.

[10] LiuB, CuiL, DengZ Protocols for the assembly and annotation of snake genomes V.2. protocols.io. 2023; 10.17504/protocols.io.5jyl8j6e9g2w/v2.

[11] LiuB, ShiY, YuanJ Estimation of genomic characteristics by analyzing *k*-mer frequency in de novo genome projects. aRxiv. 2013; 10.48550/arXiv.1308.2012.

[12] LiuB, CuiL, DengZ The genome assembly and annotation of the many-banded krait, *Bungarus multicinctus* . Gigabyte, 2023; gigabyte82. doi:10.46471/gigabyte.82.37404266PMC10315667

[13] EmmsDM, KellyS. OrthoFinder: solving fundamental biases in whole genome comparisons dramatically improves orthogroup inference accuracy. Genome Biol., 2015; 16(1): 157. doi:10.1186/s13059-015-0721-2.26243257PMC4531804

[14] VidalN, HedgesSB. The molecular evolutionary tree of lizards, snakes, and amphisbaenians. C. R. Biol., 2009; 332(2): 129–139. doi:10.1016/j.crvi.2008.07.010.19281946

[15] GuoX, ChenF, GaoF CNSA: a data repository for archiving omics data. Database, 2020; 2020: baaa055. doi:10.1093/database/baaa055.32705130PMC7377928

[16] FengZC, LiJY, FanY CNGBdb: China National GeneBank DataBase. Yi Chuan (Hereditas), 2020; 42(8): 799–809. doi:10.16288/j.yczz.20-080.32952115

[17] WangX, LiuL, ZhuW Supporting data for “Genome assembly and annotation of the Sharp-nosed Pit Viper *Deinagkistrodon acutus* based on next-generation sequencing data”. GigaScience Database, 2023; 10.5524/102426.PMC1049809837711277
